# Dysautonomia in COVID-19 Patients: A Narrative Review on Clinical Course, Diagnostic and Therapeutic Strategies

**DOI:** 10.3389/fneur.2022.886609

**Published:** 2022-05-27

**Authors:** Francisco Carmona-Torre, Ane Mínguez-Olaondo, Alba López-Bravo, Beatriz Tijero, Vesselina Grozeva, Michaela Walcker, Harkaitz Azkune-Galparsoro, Adolfo López de Munain, Ana Belen Alcaide, Jorge Quiroga, Jose Luis del Pozo, Juan Carlos Gómez-Esteban

**Affiliations:** ^1^Infectious Disease Service, University Clinic of Navarra, Pamplona, Spain; ^2^COVID-19 Department, University Clinic of Navarra, Pamplona, Spain; ^3^Immune and Infectious Inflammatory Diseases Research, IdiSNA, Navarra Institute for Health Research, Pamplona, Spain; ^4^Neurology Department, Donostia University Hospital-OSAKIDETZA, San Sebastián, Spain; ^5^ATHENEA Neuroclinics, Policlínica Gipuzkoa Grupo Quironsalud, Donostia, Spain; ^6^Neuroscience Area, Biodonostia Research Institute, San Sebastián, Spain; ^7^Neurology Department, Faculty of Medicine, University of Deusto, Bilbao, Spain; ^8^Centro de Investigación Biomédica en Red sobre Enfermedades Neurodegenerativas (CIBERNED), Institute Carlos III, Madrid, Spain; ^9^Neurology Department, Hospital Reina Sofía de Tudela-OSASUNBIDEA, Tudela, Spain; ^10^Aragon Institute for Health Research (IIS-A), Zaragoza, Spain; ^11^Neurodegenerative Diseases Group Biocruces Bizkaia Health Research Institute, Barakaldo, Spain; ^12^Neurology Department, Cruces University Hospital-OSAKIDETZA, Barakaldo, Spain; ^13^Neurology Practice, Polyclinic Mladost 1, Sofia, Bulgaria; ^14^Infectious Disease Department, Donostia University Hospital-OSAKIDETZA, San Sebastián, Spain; ^15^Department of Neurosciences, University of the Basque Country (UPV/EHU), Leioa, Spain; ^16^Pulmonary Department, University Clinic of Navarra, Pamplona, Spain; ^17^Internal Medicine Department, University Clinic of Navarra, Pamplona, Spain; ^18^Centro de Investigación Biomédica en Red en Enfermedades Hepáticas y Digestivas (CIBEREHD), Institute Carlos III, Madrid, Spain

**Keywords:** dysautonomia, Post-COVID-19 condition, socioeconomic impact, orthostatic intolerance syndromes, POTS, diagnosis, management

## Abstract

**Introduction:**

On March 11, 2020, the World Health Organization sounded the COVID-19 pandemic alarm. While efforts in the first few months focused on reducing the mortality of infected patients, there is increasing data on the effects of long-term infection (Post-COVID-19 condition). Among the different symptoms described after acute infection, those derived from autonomic dysfunction are especially frequent and limiting.

**Objective:**

To conduct a narrative review synthesizing current evidence of the signs and symptoms of dysautonomia in patients diagnosed with COVID-19, together with a compilation of available treatment guidelines.

**Results:**

Autonomic dysfunction associated with SARS-CoV-2 infection occurs at different temporal stages. Some of the proposed pathophysiological mechanisms include direct tissue damage, immune dysregulation, hormonal disturbances, elevated cytokine levels, and persistent low-grade infection. Acute autonomic dysfunction has a direct impact on the mortality risk, given its repercussions on the respiratory, cardiovascular, and neurological systems. Iatrogenic autonomic dysfunction is a side effect caused by the drugs used and/or admission to the intensive care unit. Finally, late dysautonomia occurs in 2.5% of patients with Post-COVID-19 condition. While orthostatic hypotension and neurally-mediated syncope should be considered, postural orthostatic tachycardia syndrome (POTS) appears to be the most common autonomic phenotype among these patients. A review of diagnostic and treatment guidelines focused on each type of dysautonomic condition was done.

**Conclusion:**

Symptoms deriving from autonomic dysfunction involvement are common in those affected by COVID-19. These symptoms have a great impact on the quality of life both in the short and medium to long term. A better understanding of the pathophysiological mechanisms of Post-COVID manifestations that affect the autonomic nervous system, and targeted therapeutic management could help reduce the sequelae of COVID-19, especially if we act in the earliest phases of the disease.

## Introduction

Severe acute respiratory syndrome coronavirus 2 (SARS-CoV-2) has created a pandemic, generally known as the coronavirus disease 2019 (COVID-19) pandemic, with devastating effect on the health and economy of the entire world population. The first cases of COVID-19 were reported in Wuhan, China, in November 2019, and the first cases in North America and Europe in January 2020 (1). By February 2022, more than 430 million confirmed cases of COVID-19 and more than 5.9 million deaths had been reported to the World Health Organization (WHO) (https://covid19.who.int/, as of February 27, 2022). About 80% of COVID-19 cases are paucisymptomatic and mild, and many patients recover within 2–4 weeks. However, severe pneumonia and critical multi-organ failure may occur in 15 and 5% of cases, respectively (2). Although there is a wealth of information on the clinical manifestations, therapeutic management and short-term consequences of the infection, there is less information on the residual symptoms that occur and persist in patients who have overcome acute infection but experience long-term multiorgan complications (3). These manifestations were detected from the very outset of the pandemic, and indeed, the existence of persistent symptoms (i.e., long COVID-19) after acute infection has been noted since April 2020 (4). Post-COVID symptoms are very heterogeneous and affect and involve multiple systems. Numerous pathophysiological mechanisms have been proposed that include, but are not limited to, direct or indirect invasion of the virus into the brain, immune dysregulation, hormonal disturbances, elevated cytokine levels due to immune reaction leading to chronic inflammation, direct tissue damage, and persistent low-grade infection.

The actual number of those affected who manifest symptoms after the acute episode of COVID-19 is unknown; however, in a survey carried out by the UK Government Office for National Statistics in November 2020, around 20% of patients diagnosed with COVID-19 reported symptoms that persisted 5 weeks or more after acute infection, and 10% reported symptoms lasting 12 weeks or more (3).

Frequently reported residual effects of the SARS-CoV-2 virus include a wide array of pulmonary and extrapulmonary clinical manifestations, including nervous system and neurocognitive disorders, mental health disorders, cardiovascular disorders, gastrointestinal disorders, skin disorders, and signs and symptoms associated with poor general wellbeing, including malaise, fatigue, musculoskeletal pain, and reduced quality of life. The most common neurocognitive symptoms reported are difficulties concentrating, memory deficits and cognitive impairment (5). Follow-ups conducted in Germany and the United Kingdom found post–COVID-19 neuropsychiatric symptoms in 20–70% of patients, including young adults (6). Systemic and neurocognitive deficits may last only weeks but can potentially lead to lifelong disability (2).

In a prospective study conducted in 3,762 participants from 56 countries with confirmed (diagnostic/antibody-positive; 1,020) or suspected (diagnostic/antibody-negative or untested; 2,742) COVID-19, it was found that more than 91% of participants continued to have symptoms at 7-month follow-up, mainly systemic and neurologic/cognitive symptoms. The most frequent symptoms after month 6 were fatigue, Post-exertional malaise, and cognitive dysfunction. Relapse or recurrence of symptoms, triggered primarily by exercise, physical or mental activity, and stress (7), were experienced by 85.9% of participants (95% CI, 84.8–87.0%).

In individuals at low risk of COVID-19 mortality with ongoing symptoms, 70% have impairment in one or more organs 4 months after the initial COVID-19 symptoms, including the heart (26%), lungs (11%), kidneys (4%), liver (28%), pancreas (40%) and spleen (4%) (8). Furthermore, persistent symptoms (>6 weeks) have been reported in 19% of 39 fully vaccinated healthcare workers after breakthrough infections (9).

Some studies indicate that disease severity correlates with worse and more prolonged neurologic symptoms (10, 11), while other studies have found no such correlation (5, 12).

Several symptoms are impacted by the autonomic nervous system, with fatigue described as one of the major clinical features of dysautonomia in patients with COVID-19 (13). Dysautonomia has been broadly defined as a condition where changes in the functioning of one or more components of the autonomic nervous system adversely affect health (14). Despite its prevalence, the relationship between Post-COVID symptoms and dysautonomic features has not been well-studied.

### Changes in the Criteria for the Classification of the Symptomatic Phases of COVID-19

The United Kingdom National Institute for Health and Care Excellence (NICE) (15) has defined several symptomatic phases of COVID-19 useful for the conduction and comparison of different studies, and established the following operational definitions based on the timing of signs and symptoms after illness onset. These are as follows:

- *Acute COVID-19:* signs and symptoms present up to 4 weeks after illness onset.
- *Ongoing symptomatic COVID-19*: signs and symptoms of COVID-19 persist from 4 to 12 weeks after illness onset.
- *Post-COVID-19 syndrome*: signs and symptoms that develop during or after an infection compatible with COVID-19, continue beyond 12 weeks and are not explained by an alternative diagnosis once active infection or reinfection has been ruled out.
- *Prolonged COVID/“long-COVID”/“Post-acute sequelae” of COVID (PASC)* includes both ongoing symptomatic COVID-19 (4–12 weeks) and Post-COVID-19 syndrome (12 weeks or more).

Diagnostic criteria for Post-acute phase sequelae (PASC) of SARS-CoV-2 infection, which may affect 20–60% of patients (16), were subsequently proposed (17). The term “neuro-PASC” refers to diagnostic criteria related to neurologic sequelae, including dysautonomia mentioned above. In this context, the neurologic symptoms or development of sequelae due to SARS-CoV-2 infection persist beyond 4 weeks after the onset of acute symptoms. Subacute neuro-PASC corresponds to neurologic symptoms and abnormalities present from 4 to 12 weeks after the acute phase of COVID-19, while chronic neuro-PACS refers to neurologic symptoms and abnormalities persisting or present beyond 12 weeks and not attributable to alternative diagnoses (2).

Finally, in the last consensus communication published on 6 October 2021, the WHO using a robust Delphi methodology, published a clinical case definition of the Post-COVID-19 condition reached by Delphi consensus (18) (Box 1).

Box 1 **Clinical case definition of Post-COVID-19 condition.**

Post-COVID-19 condition occurs in individuals with a history of probable or confirmed SARS-CoV-2 infection, usually 3 months from the onset of COVID-19 with symptoms that last for at least 2 months and cannot be explained by an alternative diagnosis. Common symptoms include, but are not limited to, fatigue, shortness of breath, and cognitive dysfunction, and generally have an impact on everyday functioning. Symptoms might be new onset following initial recovery from an acute COVID-19 episode or persist from the initial illness. Symptoms might also fluctuate or relapse over time. A separate definition might be applicable for children.

*Notes:*

There is no minimum number of symptoms required for diagnosis; symptoms involving different organs systems and clusters have been described.

Despite these efforts to define the picture, there is a clear need at the neurologic level to acquire a better understanding of the underlying pathophysiology of these symptoms in order to improve the therapeutic management of the different clinical pictures, in which autonomic dysfunction triggered in patients after COVID-19 (19) is of special interest.

### Dysautonomia Definition

The autonomic nervous system, which innervates all organs of the body, maintains biological homeostasis at rest and in response to stress through an intricate network of central and peripheral neurons that work automatically. Autonomic disorders can manifest in a variety of ways: deafferentation of the central autonomic centers can alter the degree or timing of peripheral autonomic effectors; autonomic efferent neuron lesions can reduce or suppress autonomic responses; and drugs or antibodies acting on autonomic neuron receptors can produce a variety of physiological phenomena ranging from hyperfunction to hypofunction and loss of function (20).

Since the autonomic nervous system is an integrative system, the clinical approach to autonomic disorders should be holistic. Rarely does the patient present with a single, clearly explained and easily identifiable symptom (20).

The search for autonomic disorders requires a careful and thorough medical history. The goals of the assessment are to identify whether there is an autonomic disorder, to locate and define its distribution, and to measure its severity. It is especially important to detect severe and treatable disorders (20).

### Socio-Economic Impact of Autonomic Dysfunction in COVID-19

The number of people with the Post-COVID-19 condition remains uncertain. Recent reports indicate that ~20–60% of COVID-19 patients experience persistent symptoms, as stated above (16), which means that between 86 and 258 million of the more than 430 million confirmed cases of COVID-19 (https://covid19.who.int/, as of February 27, 2022) would have persistent symptoms. This gives us an idea of the enormous impact of the Post-COVID-19 condition. Assuming dysautonomic symptoms occur in 2.5% of Post-COVID-19 patients (21, 22), ~2.15–6.45 million people experience Post-COVID-19 dysautonomia worldwide. This chilling statistic gives some idea of the significance of this symptomatology and of the need to deepen our understanding of it.

The socio-economic impact of COVID-19 is largely related to the development of Post-COVID fatigue, autonomic and neurohemodynamic impairment (13). The potential scale of Post-COVID-19 syndrome in lower-risk individuals, who represent up to 80% of the population, calls for urgent policies in all countries to monitor and treat the long-term implications of COVID-19 and to mitigate its impact on healthcare utilization and the economy (8). According to data from an online survey of people with suspected and confirmed COVID-19, at 7 months after suspected or confirmed COVID-19 infection, 45.2% of patients required a reduction in working hours and 22.3% were not working due to illness (7).

There is a strong association between fatigue and Post-COVID anxiety, even in the absence of a preexisting diagnosis of depression or anxiety (23).

Little is known about the prognosis of postural orthostatic tachycardia syndrome (POTS). It is estimated that about 80% of all patients with POTS improved and 60% had minimal residual symptoms during ~5 years (24). Addressing the needs of Post-COVID-19 patients will therefore require a significant investment in resources and funding both for clinical care and research. Action is needed during this window of opportunity in the interest of reducing or shortening the impact of symptoms in these patients (25) and thus promoting their earliest possible social and occupational reintegration.

This article reviews the available scientific evidence on dysautonomic symptoms during the disease, as well as the evidence on the management of the most prevalent syndromes.

## Methodology

Methodological differences in the assessment of dysautonomia in the different published studies, as well as the variations in study design, prevent us from comparing them directly. We have performed a critical narrative review with a synthesis of the current publications on the subject.

We conducted a Non-systematic literature search of the PubMed database in January 2022 for published manuscripts on dysautonomia and COVID-19. The research strategy included the key terms “dysautonomia AND COVID-19” and “autonomic symptoms AND COVID-19”. Six of the authors (F C-T, A M-O, A L-B, BT, VG, MW) independently reviewed the publications and selected those that met the inclusion criteria. Duplicate publications were removed by manual checking. Studies eligible for inclusion were all types of articles published in English or Spanish, human-centered, with well-defined COVID-19-related descriptions of dysautonomic signs and symptoms.

**Figure 1:**
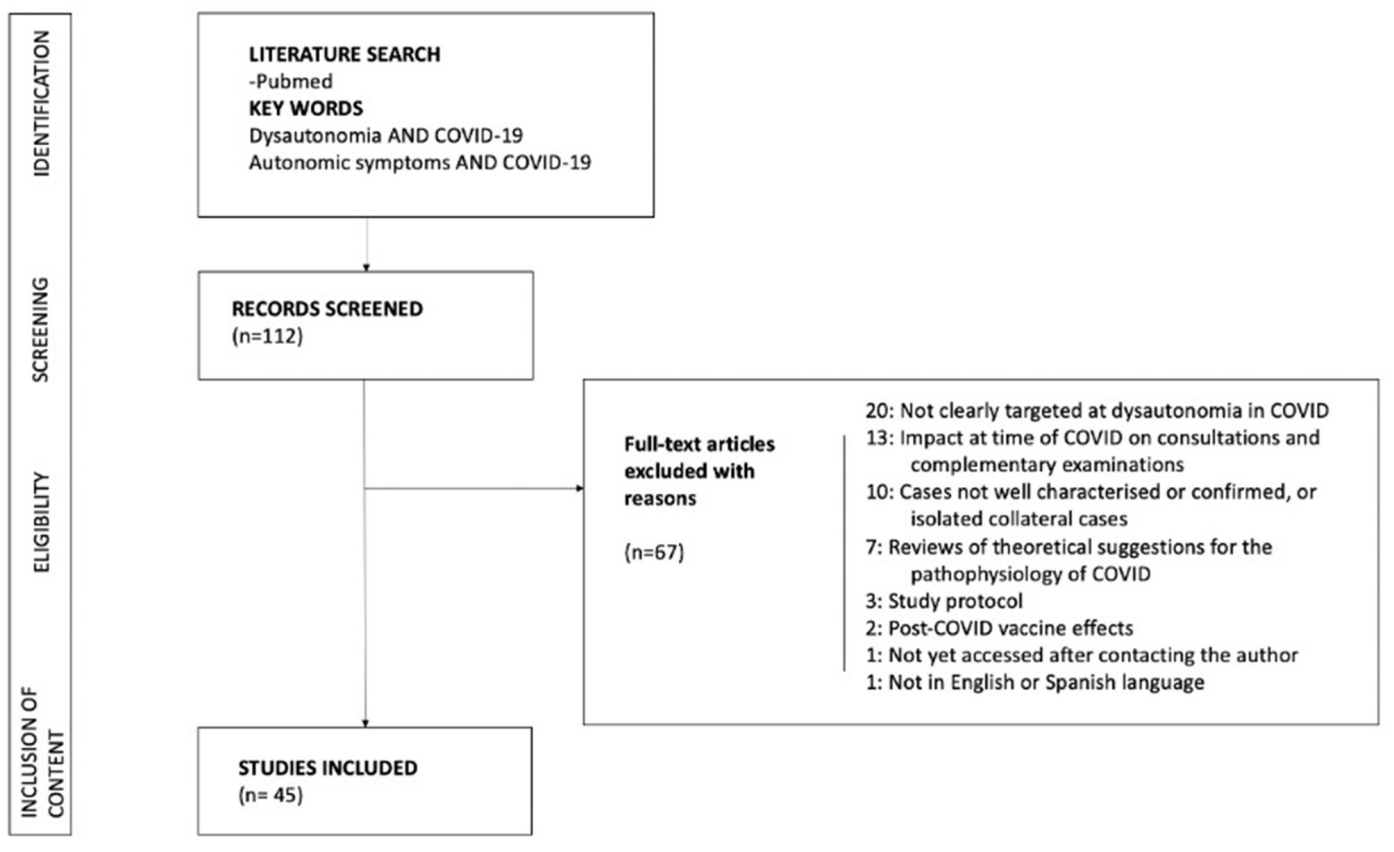
Flow chart of the study.

**Figure 2:**
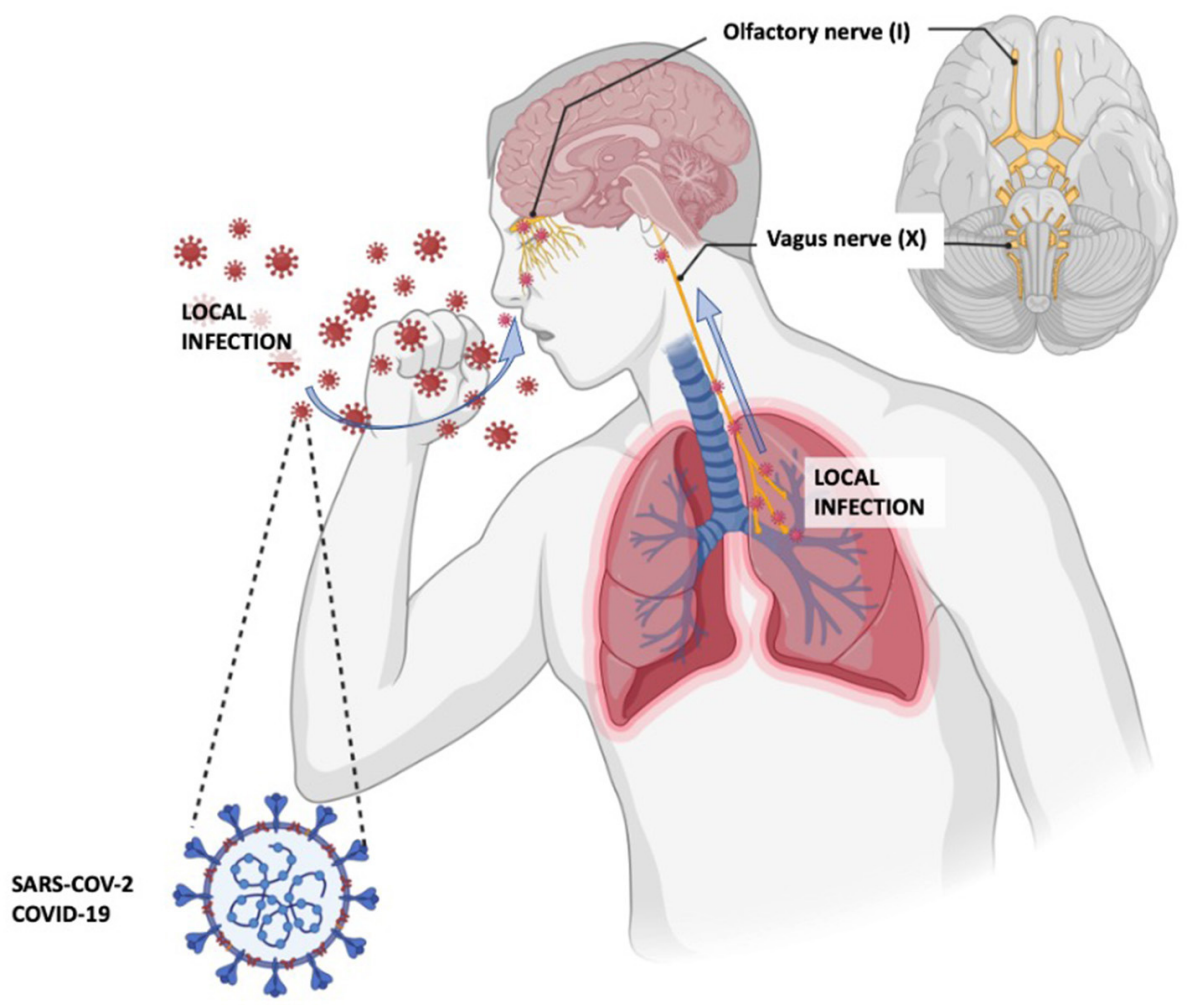
Proposed COVID-19 pathways to the central nervous system. Adapted from the article by Yachou Y et al. (26).

**Figure 3:**
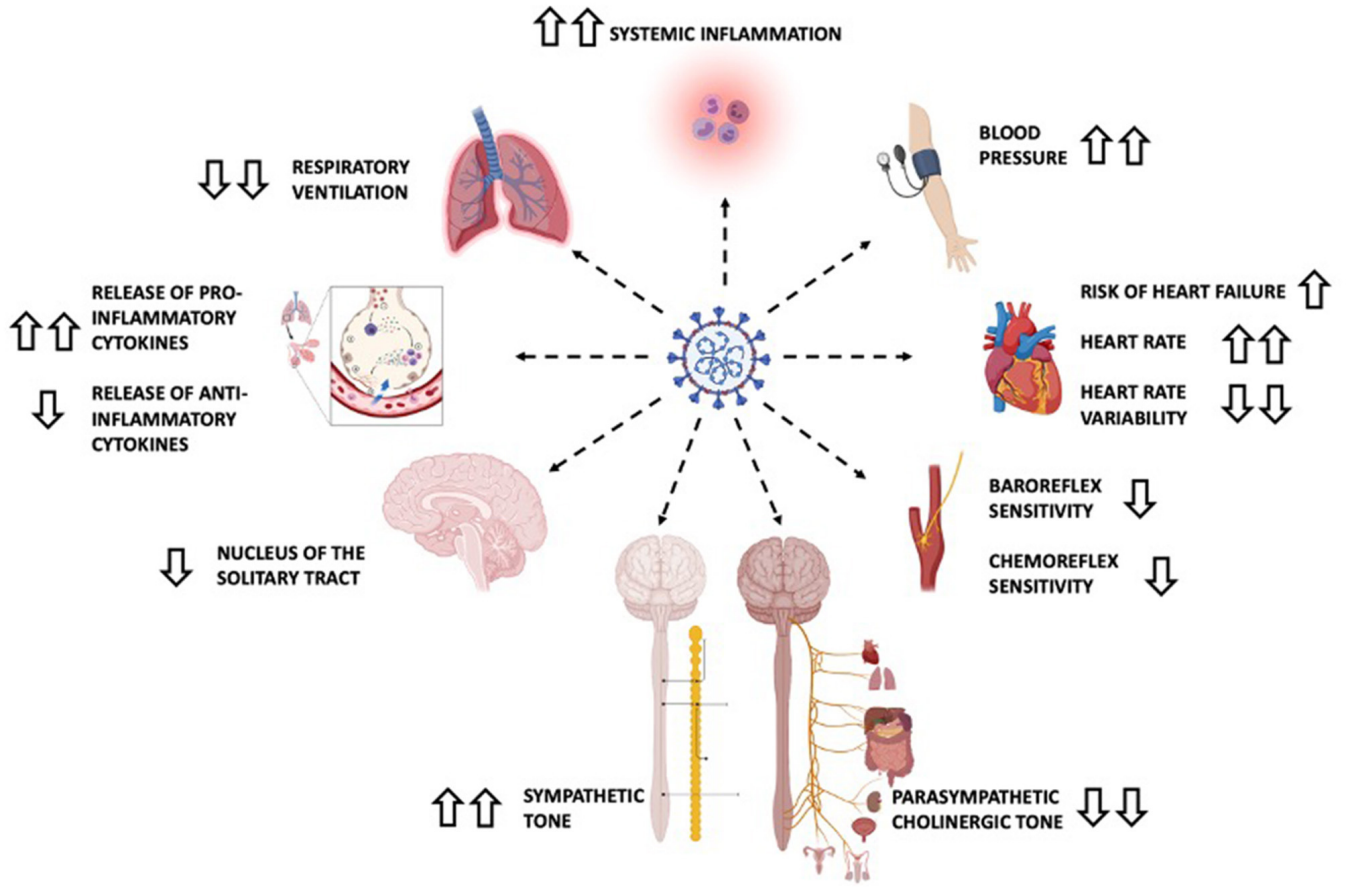
Main dysautonomic changes in severe COVID-19 infection. Information extracted from the text and based on the article by Rangon et al. (27). Upward and downward pointing arrows indicate increase and decrease, respectively. Double arrows indicate important variations. Recording hemodynamic changes and detailed neurologic examinations are both standard clinical practice but are of the utmost importance in patients with COVID-19 and manifestations suggestive of autonomic dysfunction.

Studies lacking a clear description of the diagnostic criteria for dysautonomia or COVID-19 were excluded. Study protocols, publications that did not specifically mention dysautonomia or did not focus on dysautonomia in COVID-19, as well as those published in a language other than English or Spanish were excluded (Figure 1). The text has been completed with publications obtained from PubMed that were considered relevant. Figures 2, 3 were prepared using the BioRender.com tool.

## Possible Routes of Entry of the Virus

It is thought that part of the neurologic symptomatology may be due to invasion of the central nervous system (CNS) by SARS-CoV-2. The same hypothesis was considered in the 1918 influenza pandemic when an association between influenza, encephalitis lethargica, and postencephalitic parkinsonism was observed (28). It is known that SARS-CoV-2 penetrates the olfactory mucosa, causing loss of smell, and may invade the brain tissue by migrating from the cribriform plate along the olfactory tract, or by the vagal or trigeminal pathways (26, 29) (Figure 2).

Another hypothesis is that the virus could cross the blood-brain barrier (BBB) which is disrupted or becomes more permeable through the action of inflammatory cytokines and/or monocytes (30). The virus can reach the brain tissue through the circumventricular organs (midline structures around the third and fourth ventricles).

Once inside the CNS, SARS-CoV-2-related neuronal damage can be induced either by direct cell invasion, mediated by a virus protein binding to the endothelial acetylcholine receptor, or by a cytokine-mediated dysimmune mechanism (31). Low-grade inflammation in small vessels is also thought to play a role (32). This is most likely facilitated by the inflammatory reflex and the autonomic brainstem reflex (27, 33).

## The Role of Dysautonomia in the Clinical Course of COVID-19

COVID-19 is especially life-threatening in the elderly and in those with any of a variety of chronic medical conditions. Pneumonitis and pulmonary dysfunction usually dominate the clinical picture, but it is clear that COVID-19 significantly affects other body organ systems, including the heart, gut, kidneys, and brain (14, 34). One hypothesis contends that this heightened risk may be caused by the development of dysautonomia (14).

It is not clear whether infection-associated dysautonomia is the direct result of the action of the virus on autonomic nervous system (ANS) structures or a consequence of postinfectious immune-mediated processes (34, 35).

The ANS has traditionally been viewed as consisting of the sympathetic nervous system, the parasympathetic nervous system, and the enteric nervous system. Over the past century, however, the neuroendocrine and neuroimmune systems have come to the fore, prompting a change of nomenclature to “extended autonomic system (EAS)”. Additional facets include the sympathetic adrenergic system, for which adrenaline is the key effector; the hypothalamic-pituitary-adrenocortical axis; arginine vasopressin; the renin-angiotensin-aldosterone system, with angiotensin II and aldosterone as the main effectors; and the cholinergic anti-inflammatory and sympathetic inflammasome pathways. A hierarchical brain network—the central autonomic network—regulates these systems; embedded within it are components of the Chrousos/Gold “stress system” (14).

Acute, coordinated alterations in homeostatic settings (allostasis) can be crucial for surviving stressors. Allostatic states however also increase wear and tear on both the effectors and the target organs. Intense or long-term EAS activation in the setting of chronically decreased homeostatic efficiencies (dyshomeostasis), associated with aging and chronic disorders, can prolong or intensify allostatic load, and eventually lower the thresholds for a variety of vicious cycles (positive feedback loops) that can be lethal. This phenomenon could explain the close correlation of COVID-19 mortality with age and multiple organ involvement in the disease (14).

Orthostatic intolerance, sudomotor, gastrointestinal and pupillomotor disorders are described as common complications of COVID-19, together with low tolerance for environmental conditions, and sexual dysfunction (36). Figure 3 shows the most prevalent symptoms of dysautonomia in severe COVID-19 (27).

Autonomic dysfunction associated with SARS-CoV-2 infection occurs at different temporal stages. Acute autonomic dysfunction is due to axonal damage or cardiopulmonary involvement. Iatrogenic autonomic dysfunction is a side effect caused by the drugs used and/or admission to the intensive care unit (37). Finally, late dysautonomia occurs in the *Post-COVID-19 condition*. Cardiovascular involvement, especially POTS, and to a lesser extent neurogenic orthostatic hypotension (NOH), has been more frequently observed in these latter patients. The aspects that we consider to be the most important in each phase are, in chronological order:

### Acute-Subacute Autonomic Dysfunction

Among other things, activation of the EAS in the context of acute COVID-19 increases myocardial oxygen consumption and glucose levels, depletes energy, lowers thresholds for arrhythmias, induces hypokalemia and hyponatremia, may promote renal ischemic injury and intravascular thrombosis, and can induce a form of stress cardiomyopathy. Imbalances in the inflammasome system can contribute to cytokine storms (14, 34). All these changes, facilitated by dysautonomia, generate a series of manifestations at different levels. We summarize below the neurologic, cardiovascular and respiratory manifestations.

#### Neurologic Manifestations

Like Post-Chikungunya syndrome in 2006, as well as other viral infections and vaccines, SARS-CoV-2 could trigger an immune response leading to Guillain Barré Syndrome (GBS) or other neurologic manifestations of an autoimmune nature. By the end of 2020, at least 220 patients with GBS or its variants following COVID-19 infection have been reported. GBS subtype was specified in 152 as acute inflammatory demyelinating neuropathy (AIDP), 118 cases; acute motor axonal neuropathy (AMAN) in 13; acute motor and sensory axonal neuropathy (AMSAN) in 11; Miller-Fisher Syndrome in 7; polyneuritis cranialis (PNC) in 2; and the pharyngeal-cervical-brachial variant in 1. No cases of Bickerstaff encephalitis were found (38).

It has been shown that antibodies to SARS-CoV-2 can cross-react with peripheral myelin causing GBS (39). GBS usually manifests with a florid picture of clinical dysautonomia that includes the presence of hemodynamic instability, urinary retention, gastroplegia, paralytic ileus or refractory hypertension (40).

In most patients who develop GBS, the time gap between COVID-19 infection and GBS is very short, which not only complicates treatment, but could also result in a poor prognostic clinical sign for development of severe autonomic dysfunction (41) without early detection and appropriate therapeutic management.

It is speculated that the pathogenesis of Miller Fisher syndrome following SARS-CoV-2 infection is mediated by neurotropism or aberrant activation of the immune system, with production of circulating antibodies similar to GDq1B in idiopathic Miller Fisher syndrome (42).

An increased incidence of acute motor and sensory and axonal neuropathy (AMSAN) and acute inflammatory demyelinating polyneuropathy (AIDP) is also associated with COVID-19 infection, which may present with autonomic dysfunction, especially in cases with greater axonal involvement (25, 43, 44).

Pupillary light reflex (PLR) is under the control of the autonomic nervous system. Pupil dilation is innervated by the sympathetic nervous system and pupil constriction by the parasympathetic nervous system (45). Using PRL to assess autonomic dysfunction in patients with acute COVID-19 infection varies according to the severity of clinical presentation. In an observational, cross-sectional study, higher values of pupillary dilation velocity and baseline pupil diameter were reported in 20 Non-critically-ill COVID-19 patients in the acute phase of the disease (31). Regarding critically-ill COVID-19 patients, a study in 18 patients with respiratory failure requiring mechanical ventilation for >48 h did not find, after statistical correction for possible confounders (i.e., sedation), significant differences in PLR dynamics between SARS-CoV-2-infected patients and those suffering from respiratory failure due to other causes (46).

#### Cardiovascular Manifestations

Characteristics of cardiovascular involvement in patients with COVID-19 may include myocardial lesions (myocarditis), vasculitis-like syndromes, atherothrombotic manifestations and autonomic dysfunction (47).

Maladaptive functions of the renin–angiotensin–aldosterone system (RAAS) constitute another plausible pathophysiological mechanism of SARS-CoV-2 infection–related tissue damage, probably related to central autonomic network dysfunction mentioned above. The RAAS is composed of a cascade of regulatory peptides that participate in key physiological processes in the body, including fluid and electrolyte balance, blood pressure regulation, vascular permeability, and tissue growth. Angiotensin-converting enzyme 2 (ACE2) has emerged as a potent counter-regulator of the RAAS pathway. ACE2 cleaves angiotensin I into inactive angiotensin 1–9 and degrades angiotensin II to angiotensin 1–7, which has vasodilatory, antiproliferative, antifibrotic, anti-inflammatory and sympathoinhibitory effects through binding to the Mas receptor (14, 48–50). Angiotensin II promotes vasoconstriction, fibrosis, hypertrophy and inflammation by binding to angiotensin II receptor type 1 (AT1-R) and mediating sympathoexcitation. Internalization of SARS-CoV-2 leads to inhibition of ACE2 activity and progressive depletion of membrane-bound ACE2, with ACE1/ACE2 imbalance and increased angiotensin II (50).

Fluctuating blood pressure could be explained by acute dysautonomia secondary to afferent baroreflex failure, a syndrome characterized by very labile blood pressures in which severe hypertensive crises alternate with hypotensive episodes. This phenomenon has previously been observed as a consequence of radiation therapy of the cervical region or more rarely after surgery for *Glomus caroticum* or brainstem tumors (51, 52). SARS-CoV-2 is known to have a tropism for the medullary structures of the CNS, including the ventrolateral part and the nucleus tractus solitarius, where the ACE2 receptor is highly expressed (53).

The pathophysiology of COVID-19–related myocarditis is thought to be a combination of direct viral injury and cardiac damage due to the host immune response (54). In addition, toxic effects of endogenous or exogenous catecholamines may show a pattern of Takotsubo cardiomyopathy (14).

Cardiac arrhythmias, including new-onset atrial fibrillation, heart block, and ventricular arrhythmias, are prevalent, occurring in 17% of hospitalized patients and 44% of patients in the ICU setting (34). Atrial arrhythmias are more common among patients who required mechanical ventilation than among those who did not (17.7 vs. 1.9%) (34).

#### Respiratory Manifestations

Impaired exercise tolerance is multifactorial and related to cardiac sympathetic predominance, decreased response to both sympathetic and parasympathetic stimuli that alter cardiovascular and pulmonary function, muscle tone, and impaired exercise tolerance (55). Airway sensory receptors channel information to the central nervous system, which regulates breathing and other parameters of lung function. This degree of crosstalk is achieved through three distinct airway receptors: C-fiber receptors, rapidly adapting receptors, and slowly adapting receptors. There are also deflation-activated receptors (mechano-receptors) (55).

The parasympathetic nervous system regulates pulmonary mucous production, airway smooth muscle tone, ciliary motility and transport, mucous secretion, cough reflexes and also local pulmonary inflammation and immunity (55).

SARS-CoV-2 infection-induced stress can activate the sympathetic nervous system (SNS) leading to neurohormonal stimulation and activation of pro-inflammatory cytokines with further development of sympathetic storm. Sympathetic overactivation in COVID-19 is correlated with increase in capillary pulmonary leakage, alveolar damage, and development of acute respiratory distress syndrome (56). However, it is very likely that respiratory distress is not only the result of inflammation and structural lung damage, but also of damage caused by the virus to the respiratory centers of the brain, making the management of these patients difficult (26).

The exact pathophysiological mechanism behind the **“happy” hypoxemia** phenomenon is still unknown. Patients with severe glossopharyngeal or vagus nerve lesions due to neck tumors or congenital neuropathies have reported a disconnect between the perceived degree of hypoxia and dyspnea after the development of pneumonia. Possible damage to hypoxia-sensitive afferent neurons in persons with COVID-19 could be due to cytokine storm or the direct effect of SARS-CoV-2 on mitochondria or nerve fibers. The brain magnetic resonance imaging (MRI) findings and pathoanatomic studies in fatal cases of COVID-19 are so far inconsistent in this regard and do not provide a pathophysiological correlate to satisfactorily explain the absence of dyspnea in these patients (57).

#### Other Manifestations

The incidence of gastrointestinal manifestations has ranged from 12 to 61% in patients with COVID-19 (34). In a recent meta-analysis of 29 studies, the pooled prevalence of individual symptoms was reported to include that of anorexia (21%), nausea and/or vomiting (7%), diarrhea (9%), and abdominal pain (3%) (58).

In hospitalized COVID-19 patients, hypokalemia is frequent and is also associated with increased mortality. Low serum potassium may reflect increased aldosterone-mediated sodium/ potassium exchange in the kidneys, as well as endogenous and exogenously administered epinephrine (14). ACE2 expression has been reported in the endocrine pancreas, albeit inconsistently. Direct binding of SARS-CoV-2 to ACE2 on β-cells could contribute to insulin deficiency and hyperglycemia. The increase in counterregulatory hormones that contributes to hepatic glucose production, decreased insulin secretion, ketogenesis and insulin resistance (34) promotes hyperglycemia in patients with COVID-19 at the time of hospitalization and has been related to adverse prognosis (14).

### Drug-Induced Autonomic Impairment and/or ICU Admission

Cardiac arrhythmias, including atrial fibrillation and life-threatening atrioventricular block, can be induced by drugs used in the treatment of COVID-19. Among the drugs used, especially at the beginning of the pandemic, are chloroquine/hydroxychloroquine, macrolides (azithromycin) and quinolones that can cause *Torsades de Pointes*-type arrhythmias or other lethal arrhythmias as a potential consequence of QT prolongation. Other drugs with arrhythmogenic potential include other antiviral agents such as lopinavir/ritonavir, favipiravir, immunomodulatory treatments as tocilizumab, fingolimod, the anesthetic propofol, the antiemetic domperidone, class IA and III antiarrhythmics and the antipsychotic haloperidol, used in the initial phases of the pandemic mainly to combat the so-called cytokine storm. Drug combinations, especially QT-prolonging agents, used in the early stages may have induced increased arrhythmogenicity and secondary lethality (59). We suspect that all these events are facilitated by disorders in the autonomic system.

Many critically ill COVID-19 patients often have previous comorbidities, which together with acute comorbidities such as electrolyte disturbances (hypokalemia, hypomagnesemia), fever, systemic inflammation and excess autonomic lability contributes to increased cardiovascular morbidity and mortality (47, 59).

### Dysautonomia in Post-COVID-19 Condition

Dysautonomic symptoms observed after SARS-CoV-2 infection are similar to those described after other viral infections such as mumps, human immunodeficiency virus, hepatitis C, Epstein-Barr, or Coxsackie type B virus (60, 61). In the severe acute respiratory syndrome (SARS) epidemic of 2002–2004 [8,422 cases and 916 deaths (11% mortality)], one study reported that 40% of patients (67% female) still had chronic fatigue nearly 2 years after infection (62). Studies utilizing autonomic reflex testing in post-SARS syndrome are scarce. One study of 14 patients (85% female) demonstrated an abnormal 30:15 ratio on active stand testing in 4/14 (29%) patients at 6 months Post-infection, with three reporting orthostatic intolerance (63). The 2012 coronavirus epidemic caused by the Middle East respiratory syndrome virus (MERS) was more limited and resulted in 2,468 cases and 851 deaths (34% mortality), but to our knowledge there are no reports of autonomic impairment following MERS (1).

Approximately 2.5% of patients with infection suffered Post-COVID-19 autonomic dysfunction (21, 22). In an observational cohort study involving 205 patients with confirmed or probable COVID-19 infection who met specific eligibility criteria (hospitalization, life-limiting symptoms beyond 12 weeks, desaturation < =95% on a Harvard step test, or chest pain with electrocardiographic changes during acute illness) a high prevalence (25%) of Post-COVID dysautonomia (64) was shown. Dysautonomia was defined as a resting heart rate (HR) >75 bpm, HR increase with exercise <89 bpm, and HR recovery <25 bpm 1 min after exercise (64) and was associated with objective functional limitations (reduced work rate and peak oxygen consumption and a steeper V_E_/V_CO2_ slope), but was not associated with subjective symptoms or limitations (64).

#### Orthostatic Intolerance Syndromes

It has been proposed that some symptoms of the *post COVID-19 condition* may be related to a virus- or immune-mediated disruption to the autonomic nervous system, resulting in transient or long-term orthostatic intolerance syndromes. It is well established that some cases of autonomic disorders such as NOH and POTS are associated with autoantibodies against α-/β-adrenoceptors and muscarinic receptors (65–70).

When a healthy person stands upright, blood pools in the pelvis and legs, reducing venous return to the heart. This is detected by cardiac and aortic baroreceptors, which respond by increasing sympathetic and adrenergic tone (mediated by noradrenaline and epinephrine/adrenaline, respectively). This results in tachycardia to compensate for the reduction in stroke volume and is followed by vasoconstriction in the splanchnic vascular bed, which increases the venous return to the heart (69).

Orthostatic intolerance is the inability to tolerate the upright posture because of symptoms of cerebral hypoperfusion or sympathetic activation, or both, which are relieved by recumbency (71). In orthostatic intolerance, the release of epinephrine and norepinephrine causes pronounced tachycardia, which is experienced as palpitations, breathlessness, and chest pain. Very high catecholamine levels can lead to paradoxical vasodilatation, sympathetic activity withdrawal and activation of the vagus nerve, resulting in hypotension, dizziness and ultimately, syncope (69).

Orthostatic intolerance syndromes include neurogenic orthostatic hypotension, neuromediated syncope and postural orthostatic tachycardia syndrome, and even orthostatic hypotension and neurally-mediated syncope should be considered. Since POTS appears to be the most common autonomic phenotype among PACS patients (1), the explanation of this feature has been expanded.

##### Neurogenic Orthostatic Hypotension (NOH)

Defined as a reduction of systolic blood pressure of at least 20 mmHg or a reduction in diastolic blood pressure of at least 10 mmHg within 3 min of active standing or head-up tilt on a tilt table (71–73). Approximately one third of persistent orthostatic hypotension is neurogenic (20). It is due to reduced norepinephrine release from postganglionic sympathetic nerves, resulting in defective vasoconstriction when assuming the upright position. It is most frequent in patients with diabetes mellitus, neurodegenerative disorders and small fiber neuropathies (73).

##### Neuromediated Syncope (Particularly Vasovagal Syncope)

This is the most common cause of syncope. The median number of episodes over a lifetime is 3, with a recurrence rate of 30% at 30 months (74).

##### Postural Orthostatic Tachycardia Syndrome (POTS)

POTS is a disorder in which patients frequently experience symptoms of orthostatic intolerance in response to postural stressors, despite autonomic reflexes that are generally preserved (71). The main POTS mechanisms are impaired sympathetically- mediated vasoconstriction in the lower limbs (neuropathic POTS), excessive cardiac sympathoexcitation response (hyperadrenergic POTS), volume dysregulation, joint hypermobility, and physical deconditioning. POTS is characterized by an increase of 30 bpm or more over baseline or a sustained heart rate of more than 120 bpm, according to current standing criteria, and symptoms associated with orthostatic intolerance without a drop in blood pressure (Appendix 1) (71). There may be an overlap between POTS and other disorders, in particular, orthostatic hypotension, vasovagal syncope, panic disorders, psychogenic pseudosyncope, chronic fatigue syndrome, Ehlers–Danlos syndrome, mast cell activation disorder and cardiac arrhythmias and should be considered in complex cases (71, 75).

Symptoms of cerebral hypoperfusion that may occur with any of the disorders of orthostatic intolerance include lightheadedness, dizziness, presyncope, vision and hearing changes, lower limb or generalized weakness, and cognitive difficulties (often vaguely termed brain fog). Symptoms of sympathoexcitation that distinguish POTS from orthostatic hypotension include palpitations, chest pain, dyspnea, tremulousness, sweating, pallor, nausea, diarrhea, and coldness of the extremities (Appendix 2) (76).

The affected population is usually young and predominantly female (77). Prevalence estimates are imprecise and there are no European data available to our knowledge. In the USA, estimates range from 0.2 to 1.0% in the general population. The onset of POTS may be precipitated by typical immunological stressors such as viral infection (20–50% of patients), frequently of the upper respiratory or gastrointestinal tract, vaccination (70), trauma, pregnancy, surgery, cardiovascular deconditioning (78) or even after a period of intense psychosocial stress. However, in a considerable number of patients with POTS, there is no clear identifiable trigger (79). Cardiac symptoms include chest pain, palpitations, exercise intolerance and orthostatic intolerance. More than 90% of patients with POTS have at least one gastrointestinal (GI) symptom, with nausea, abdominal pain, and bloating being the most common (80). Other symptoms that frequently accompany POTS include fatigue, “mental confusion”, headache, temporomandibular joint disorder, fibromyalgia and sleep disturbances (81) and others listed by organ system in Appendix 3. It is recommended that all patients presenting with signs or symptoms of POTS should be evaluated to rule out the diagnosis of POTS (7, 82).

These syndromes may be exacerbated by hypovolemia resulting either from the initial infection or physical deconditioning following prolonged bed rest in the intensive care unit; prolonged bed rest leads to reduced cardiac output and stroke volume, hypovolemia, baroreflex dysfunction and decreased sympathetic responsiveness (69).

#### Other Symptoms

Other more Non-specific symptoms such as palpitations, tachycardia during mild exertion, “resting heart rate increase” (11%), chest pains/discomfort (16%), labile blood pressure, new-onset hypertension (1%), gastrointestinal symptoms (e.g., abdominal pain, bloating, nausea/vomiting (16%), gastroparesis, constipation or loose stools), sleep disorders (11%), flushing (5%), peripheral vasoconstriction, sweating abnormalities (17%), temperature intolerance and even unexplained low-grade fever are also thought to be due to autonomic dysfunction (1, 83) and their presence in any person after SARS CoV2 infection should prompt a thorough examination for possible autonomic dysfunction (69).

Sinus tachycardia, episodic sinus bradycardia and sinus pauses have been described as manifestations of autonomic dysfunction in patients with COVID-19 infection (84). Pupillary responses were impaired in patients recovering from COVID-19 vs. healthy controls, showing a larger resting-state pupil diameter and higher pupil contraction velocity, and lower values of dilatation latency and duration of pupil constriction (45).

## Detection and Diagnosis of Dysautonomia in COVID-19

A very detailed anamnesis with a thorough medical history is essential to obtain all the necessary information from the patient. Therefore, questions should be asked about different aspects. Frequent comorbidities include migraine and other headaches, inappropriate sinus tachycardia, visceral hypersensitivity, gastrointestinal dysmotility, chronic fatigue, insomnia, fibromyalgia, and often autoimmune diseases (71, 85). The histories of SARS-CoV-2 survivors with persistent autonomic dysfunction may reveal frequent episodes of fainting, dizziness, lightheadedness, and/or palpitations, revealing underlying hypotensive susceptibility or prior orthostatic intolerance syndrome (85, 86). Unexplained dyspnea, fatigue, chest pain, persistent dizziness, diarrhea, recurrent presyncope episodes, anxiety, panic attacks with low-threshold emotional triggers or symptoms of irritable bowel syndrome, among others, have been observed (69, 71). These patterns may be explained by autonomic instability and may be a consequence of deconditioning, hypovolemia and immune-mediated or viral neuropathy (69). Although the exact etiology is unknown, it is thought that patients with dysautonomia have a less favorable body composition compared to those without dysautonomia (higher body mass index and waist circumference) (64).

The quantity and diversity of the symptoms mentioned are the reason why, among the proposed Neuro-PASC diagnostic criteria, orthostatic intolerance and cardiovascular, respiratory, gastrointestinal, and genitourinary manifestations are regarded as significant and related to autonomic dysfunction (2).

In Post-COVID patients with suspected dysautonomia, the Composite Autonomic Symptom Scale 31 (COMPASS-31) questionnaire is a sensitive tool to test the likelihood of autonomic dysfunction. This questionnaire has been previously applied to COVID-19 survivors, who showed significantly higher scores than controls, with an optimal cut-point for ruling out cardiovascular autonomic dysfunction of 13.25 (85).

Parameters related to **heart rate variability and blood pressure** in sitting and standing positions seem to be another key element in the detection of dysautonomia in patients with COVID-19 (20). Sinus tachycardia, episodic sinus bradycardia, and sinus pauses have been described as autonomic dysfunction manifestations in patients with COVID-19 infection (84). Table 1 offers a simple guideline for monitoring blood pressure and heart rate in this context.

**Table 1:** Interpretation of blood pressure and heart rate measurements in the event of clinical suspicion of orthostatic hypotension (20) and after differential diagnosis with vertigo, postural instability, ataxia, weakness of leg muscles, and osteoarthritis with weight-bearing musculoskeletal pain.

**After 5^**′**^supine position**	***1st measurement***	**If BP: >140/90 mmHg → Probable NOH**
**1** **′** **standing**	*2nd measurement*	If BP: ↓*c*+20/10 mmHg → **hTO** [If BP: ↓+30/15 mmHg → assess whether MSA phenotype exists]
**2** **′** **-5** **′** **standing**	*3rd−4th measurements*	In case of high clinical suspicion without objective proof of hTO in the measurements, carry out several repetitions in this range until: - BP: ↓+20/10 mmHg → **hTO** [if also not so pronounced HR ↑ → **NOH**]
**10′standing** **(or head up tilt)**	*5th measurement*	Sustained HR ↑+30 lpm* without hTO → **POTS**

*hTO, Orthostatic hypotension; NOH, neurogenic orthostatic hypotension; BP, blood pressure (mmHg); MSA, multiple system atrophy; HR, heart rate; POTS, Postural orthostatic tachycardia syndrome; ↓, decrease; ↑, increase*.

^*^For individuals between 12 and 19 years old, ↑+40 lpm is required.

Blood tests (including complete blood count, renal function, B-type natriuretic peptide, electrolytes, thyroid stimulating hormone, and morning cortisol), resting 12-lead ECG, and the 6-min walking test should be routinely evaluated (85).

Holter ECG monitoring, 24 h ambulatory blood pressure monitoring, cardiothoracic imaging (chest X-ray, chest Computed Tomography, echocardiography, and cardiac magnetic resonance) and exercise testing are also invaluable diagnostic tools for the study of Post-acute sequelae of SARS-CoV-2 and COVID-19 complications (61, 85). It has been suggested that remote electrophysiological monitoring or long-term telemonitoring could be a very useful tracking tool after hospital discharge, especially in patients who have been critically ill (47, 59).

Active standing and/or head-up tilt tests are very useful for evaluating PASC patients, especially in individuals with inappropriate/orthostatic tachycardia, unexplained syncope, or syndromes of orthostatic intolerance. Other autonomic function tests include the Valsalva maneuver, deep breathing, and sweat function testing (85). Sudomotor function is an indirect index of sympathetic cholinergic Non-myelinated C-fiber activity, since sweat glands lack parasympathetic innervation (31). It can be assessed using *Sudoscan*, which allows estimation of electrochemical skin conductance (ESC) (31, 87). Abnormal ESC results suggest autonomic small fiber neuropathy and require confirmation with other validated techniques of sudomotor function, such as QSART testing and skin biopsy (1).

A feature of PASC patients with POTS-like symptoms is a high prevalence of specific circulating autoantibodies, including G-protein-coupled receptor (GPCR) antibodies (such as adrenergic, muscarinic and angiotensin II type-1 receptors) and the ganglionic neuronal nicotinic acetylcholine receptor (g-AChR) (75). Other recognized autoantibodies in POTS include circulating anti-nuclear, anti-thyroid, anti-NMDA-type glutamate receptor, anti-cardiac protein, anti-phospholipid, and Sjögren's antibodies (61, 71, 75, 85). Although neither sensitive nor specific, autoantibody testing can be helpful in selected cases. Specific tests for mast cell activation syndrome may also be considered in PASC patients with flushing episodes, frequent headaches, and persistent gastrointestinal symptoms (85).

**Figure 4:**
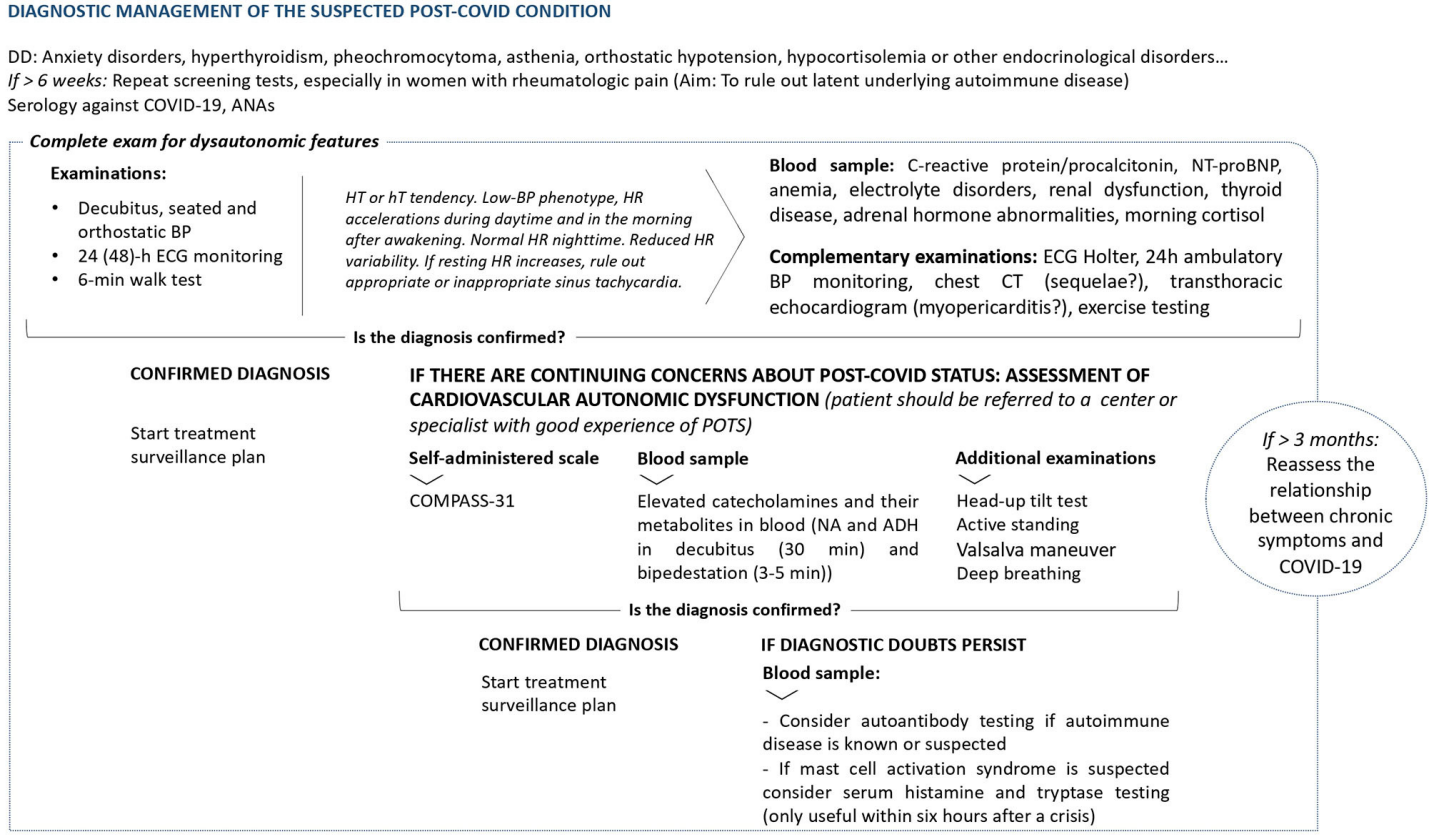
General indications for examining chronic symptoms described after COVID-19 (61, 75, 85). DD, Differential diagnosis; ANAs, antinuclear antibodies; BP, Blood pressure; h, hours; ECG, electrocardiogram; min, minutes; HT, Hypertensive; hT, Hypotensive; HR, Heart Rate; NT-proBNP, N-terminal prohormone of brain natriuretic peptide; CT, Computed Tomography; POTS, postural orthostatic tachycardia syndrome; COMPASS-31, Composite Autonomic Symptom Scale 31 questionnaire; NA, Noradrenaline; ADH, Vasopressin.

Schematic summary of the diagnostic management of a suspected Post-COVID condition is shown in Figure 4.

## Possible Treatments for Dysautonomia in COVID-19

### Cardiovascular Dysautonomic Involvement

When there is cardiovascular dysautonomia, the following order is recommended (55, 85, 88).

1/Physical reconditioning:

◦Progressive aerobic exercise training programs.
◦Use of compression accessories.
◦Water and salt intake.
◦Drinking water before getting up in the morning.
◦Sleeping with the head raised in bed.
◦Avoid situations that may exacerbate symptoms (sleep deprivation, exposure to heat, alcohol intake or large meals).

RECOMMENDATION: At the onset of prodromal symptoms and to delay/prevent vasovagal syncope, perform physical maneuvers such as crossing legs, tensing muscles and squatting.

2/ In case of insufficient or complementary Non-pharmacological measures in patients with severe refractory symptoms (Table 2).

**Table 2:** Therapeutic options in case of insufficient non-pharmacological measures or as a supplement in patients with serious refractory symptoms.

*Volume expanders*	Fludrocortisone, desmopressin, and intravenous saline
*Heart rate inhibitors*	Propranolol, ivabradine, and pyridostigmine
*Vasoconstrictors*	Midodrine, octreotide, methylphenidate, and droxidopa
*Sympatholytic drugs*	Clonidine and methyldopa

Because of the tendency for blood pressure to fluctuate, as explained above, it is important to maintain a euvolemic state and avoid excessive fluid administration during episodes of hypotension, with gradual titration of vasopressors to avoid excessive blood pressure, and to use short-acting antihypertensive drugs in hypertensive crises (89).

### Postural Orthostatic Tachycardia Syndrome

There are some more specific management protocols available to help treat POTS. The aspects mentioned below represent a summary of the nonpharmacologic and pharmacologic therapeutic options available for the management of POTS. For more detail, it is advisable to consult documents that address these issues in greater depth (90, 91).

- *Non-pharmacologic measures*: There is no Class I recommendation. The Class IIA recommendation is based on physical training to avoid chronicity of symptoms. Indications based on the management of orthostatic intolerance syndromes, such as educating the patient about the pathology, simple isometric, aerobic and resistance exercises, ensuring fluid replacement (2–3 liters of water per day, avoiding caffeine and alcohol) and one or two additional teaspoons of salt per day are maintained (Class IIB), as well as moving carefully from a lying or sitting to a standing position and avoiding exacerbating factors such as prolonged standing, hot environments and dehydration, using waist-high compression garments and assessing the need for fluid expanders if hypovolemia is considered to be a dominant symptom, among others (69, 90). Long-term repeated saline infusions are not recommended (78).
- *Pharmacologic measures:* Midodrine, beta-blockers, fludrocortisone, pyridostigmine, clonidine and alpha-methyldopa (Class IIB) (69). The only drugs that have demonstrated benefits in randomized trials are propranolol and pyridostigmine (71). Since there is no good evidence in this regard, polypharmacy is frequent; the overall effects of drug therapy however are modest. Likewise, it is recommended to discontinue the intake of noradrenaline reuptake inhibitors such as duloxetine, nortriptyline, and tapentadol. It should also be considered whether the indication of fludrocortisone, midodrine, clonidine methyldopa or propranolol is necessary, bearing in mind that these drugs are not usually very well tolerated (69, 91).

A schematic proposal for the management of orthostatic intolerance is set out in Table 3.

**Table 3:** Therapeutic proposal for orthostatic intolerance and intended effects (71, 73).

**Treatment**	**Mechanism**
**Non-pharmacologic**	
Increase water and sodium intake	Avoids hypovolemia
Compression and physical countermaneuvers	Reduces venous pooling
Physical exercise training, including gradual resistance and lower extremity resistance training	Improves physical deconditioning and reduces venous pooling
**Pharmacologic**	
Propranolol: 10 mg 1–3 times/day	Reduces standing heart rate and improves orthostatic symptoms, especially in hyperadrenergic patients with POTS
Midodrine: 2.5–15 mg 2–3 times/day (3–4 h before going to bed)	Reduces venous pooling and orthostatic hypotension, especially in neuropathic patients with POTS. Patients should be advised not to lie flat for at least 4 h after any dose of midodrine to avoid supine hypertension
Pyridostigmine: 30–60 mg 2–3 times/day	Reduces orthostatic tachycardia and improves chronic symptoms without worsening supine hypertension. Use should be limited in case of diarrhea, abdominal cramps, pain, nausea, urinary frequency and urgency
Fludrocortisone: 0.05–0.2 mg once/day	The effect only lasts 1–2 days, avoid prolonged use due to renal and cardiac involvement
Ivabradine: 5–10 mg	Reduces heart rate without affecting blood pressure
IV fluid therapy (saline)	Improves symptoms quickly although the effect lasts a short time. It is considered a bridging therapy
Others: - Droxidopa 100–600 mg 3 times/day (3–4 h before bedtime) - Atomoxetine 10–18 mg 2 times/day	

### Other Therapeutic Options

Other therapeutic options include Non-invasive neuromodulation (especially transcranial direct current stimulation, repetitive transcranial magnetic stimulation and vagus nerve stimulation) which could be used in patients with COVID-19 and autonomic dysfunction (92). It seems that it may on the one hand reduce the impact of the infection by stimulating regions involved in the regulation of systemic anti-inflammatory and/or autonomic responses, prevent neuroinflammation and aid recovery of breathing, and, on the other, improve the symptoms of musculoskeletal pain, systemic fatigue, physical and cognitive rehabilitation after the disease, even if it has been critical, as well as treat the distress generated by the disease (92).

## Key Points

- Dysautonomia, present in at least 2.5% of COVID-19 patients, is clinically similar to dysautonomia secondary to other viral infections. The prevalence of Post-COVID dysautonomia could rise to 25% in those patients who met specific eligibility criteria (hospitalization, life-limiting symptoms beyond 12 weeks and so on).
- Potentials mechanism of autonomic impairment caused by SARS CoV2 are based on direct tissue damage, immune dysregulation, hormonal disturbances, elevated cytokine levels due to immune reaction leading to chronic inflammation, and persistent low-grade infection. The EAS with allostasis and dyshomeostasis may partially explain the mortality and multi-organ involvement in COVID-19 patients.
- The major socioeconomic impact of symptom persistence after COVID-19 infection stems from fatigue, autonomic and neurohemodynamic involvement, hence the need for early intervention in these areas.
- Dysautonomic involvement secondary to COVID-19 may be acute-subacute, caused by drugs and/or ICU admission, and chronic, as in the Post-COVID-19 condition, due to orthostatic intolerance syndromes of autoimmune origin.
- A careful neurologic assessment is necessary in any patient with findings compatible with autonomic dysfunction following SARS CoV-2 infection.
- Protocols for the diagnostic and therapeutic management of autonomic dysfunction are mainly aimed at avoiding triggers of orthostatic intolerance by means of pharmacologic and Non-pharmacologic measures.

## Limitations

Some limitations need to be acknowledged in the interpretation of our results. First, this is not a systematic review. We limited our scope to selected articles which we believed could be the most representative ones. Secondly, we relied on PubMed only for our search strategy. Among the strengths of our study, we analyzed an important quantity of articles highlighting the most relevant research on COVID-19 and dysautonomia.

## Conclusion

Two years after the declaration of the COVID-19 pandemic, patients affected by this disease continue to manifest patterns of neurological involvement attributable to autonomic dysfunction. This could be the result of a multifactorial etiology deriving from physical deconditioning after time spent isolated in home, hospital wards or intensive care units, hypovolemia, virus-mediated neuropathy, or an immune response secondary to infection. One of the consequences is that a high percentage of patients with COVID-19 do not make a full return to work due to residual symptoms. The socioeconomic impact is considerable and could be significantly reduced with an appropriate diagnostic and therapeutic protocol for the underlying autonomic dysfunction. Considering the wide dissemination of COVID-19 worldwide and the extraordinary dissemination of the SARS-CoV2 omicron variant and its emerging subvariants, it would appear to be imperative to adopt measures that, in addition to containing the spread of the virus, also help improve the acute management of infected patients and prevent and/or reduce the long-term sequelae of the infection.

Among these measures, one would be improving access to autonomic testing for early diagnosis of autonomic dysfunction. This would allow early treatment, reducing the associated morbidity and mortality and thus containing its personal and socioeconomic impact.

At the same time, the sheer scale of the infection and of the Post-COVID-19 syndrome presents a unique opportunity to add to our knowledge and understanding of the specific mechanisms responsible for orthostatic intolerance, POTS-like symptoms, and their duration. It is particularly noteworthy that the comprehension of the mechanisms of self-immunity could generate new pathophysiological hypotheses applicable to other disorders with which could share clinical similarities such as chronic fatigue syndrome or fibromyalgia. This improvement in awareness may turn out to help ameliorate diagnostic accuracy in these entities, currently very diffuse and poorly managed. Hence the value of studying in depth the clinical pictures found in patients with the Post-COVID-19 syndrome, especially Post-COVID-19-POTS condition, for which a special effort is required in terms of clinical care and resources devoted to their research.

A better recognition of dysautonomia will help to improve the management of COVID-19 in all its phases, providing information for possible diagnostic and therapeutic tools applicable not only to these patients, but also to those affected by other pathologies with physiopathological similarities (other viral conditions, Alzheimer's disease, Parkinson's disease, and so on). We therefore recommend further studies to explore the prevalence, pathophysiology, clinical features, and treatment approach in patients with COVID-19-related dysautonomia.

## Author Contributions

FC-T and AM-O: literature review, data processing and analyses, interpretation of results, language editing, review and drafting of the first manuscript, and interpretation of results. AL-B, BT, VG, and MW: literature review, data processing, and interpretation of results. HA-G, AL, AA, JQ, and JP: critical revision of the manuscript. JG-E: conception and design of the article, literature review, interpretation of results, and critical revision of the manuscript. All authors contributed to the article and approved the submitted version.

## Conflict of Interest

The authors declare that the research was conducted in the absence of any commercial or financial relationships that could be construed as a potential conflict of interest.

## Publisher's Note

All claims expressed in this article are solely those of the authors and do not necessarily represent those of their affiliated organizations, or those of the publisher, the editors and the reviewers. Any product that may be evaluated in this article, or claim that may be made by its manufacturer, is not guaranteed or endorsed by the publisher.

## Abbreviations

ACE2: Angiotensin-converting enzyme 2
ADH: Vasopressin
AIDP: Acute inflammatory demyelinating neuropathy
AMAN: Acute motor axonal neuropathy
AMSAN: Acute motor and sensory axonal neuropathy
ANS: Autonomic nervous system
AT1-R: Angiotensin II receptor type 1
BBB: Blood-brain barrier
BP: Blood pressure (mmHg)
CI: Confidence Interval
CNS: Central nervous system
COMPASS-31: Composite Autonomic Symptom Scale 31
COVID-19: Coronavirus disease 2019
CT: Computed tomography
EAS: Extended autonomic system
ECG: Electrocardiogram
ESC: Electrochemical skin conductance
g-AChR: Ganglionic neuronal nicotinic acetylcholine receptor
GBS: Guillain Barré
Syndrome
GPCR: G-protein-coupled receptor
HR: Heart rate
HT: Hypertensive
hT: Hypotensive
hTO: Orthostatic hypotension
ICU: Intensive care unit
MasR: Mas receptor
MERS: Middle East respiratory syndrome
min: Minutes
MRI: Magnetic resonance imaging
MSA: Multiple system atrophy
NA: Noradrenaline
NICE: The United Kingdom National Institute for Health and Care Excellence
NOH: Neurogenic Orthostatic hypotension
NT-proBNP: N-terminal prohormone of brain natriuretic peptide
PASC: Post-acute sequelae of COVID
PLR: Pupillary light reflex
PNC: Polyneuritis cranialis
PNS: Peripheral nervous system
POTS: Postural tachycardia syndrome
QSART: Quantitative sudomotor axon reflex testing
RAAS: The renin-angiotensin-aldosterone system
SARS-CoV-2: Severe acute respiratory syndrome coronavirus 2
SARS: Severe acute respiratory syndrome
SNS: Sympathetic nervous system
WHO: World Health Organization.

## References

[1] Larsen NW Stiles LE Miglis MG. Preparing for the long-haul: autonomic complications of COVID-19. Auton Neurosci. (2021) 235:102841. 10.1016/j.autneu.2021.102841 34265539 PMC8254396

[2] Moghimi N di Napoli M Biller J Siegler JE Shekhar R McCullough LD. The neurological manifestations of post-acute sequelae of SARS-CoV-2 infection. Curr Neurol Neurosci Rep. (2021) 21:44. 10.1007/s11910-021-01130-1 34181102 PMC8237541

[3] Venkatesan P. NICE guideline on long COVID. Lancet Respir Med. (2021) 9:129–38. 10.1016/S2213-2600(21)00031-X 33453162 PMC7832375

[4] Carfi A Bernabei R Landi F Gemelli Against C-P-ACSG. Persistent symptoms in patients after acute COVID-19. JAMA. (2020) 324:603–5. 10.1001/jama.2020.12603 32644129 PMC7349096

[5] Groff D Sun A Ssentongo AE Ba DM Parsons N Poudel GR. Short-term and long-term rates of postacute sequelae of SARS-CoV-2 infection: a systematic review. JAMA Netw Open. (2021) 4:e2128568. 10.1001/jamanetworkopen.2021.28568 34643720 PMC8515212

[6] Boldrini M Canoll PD Klein RS. How COVID-19 affects the brain. JAMA Psychiatry. (2021) 78:682–3. 10.1001/jamapsychiatry.2021.0500 33769431 PMC9894299

[7] Davis HE Assaf GS McCorkell L Wei H Low RJ Re'em Y. Characterizing long COVID in an international cohort: 7 months of symptoms and their impact. EClinicalMedicine. (2021) 38:101019. 10.1016/j.eclinm.2021.101019 34308300 PMC8280690

[8] Dennis A Wamil M Alberts J Oben J Cuthbertson DJ Wootton D. Multiorgan impairment in low-risk individuals with Post-COVID-19 syndrome: a prospective, community-based study. BMJ Open. (2021) 11:e048391. 10.1136/bmjopen-2020-048391 33785495 PMC8727683

[9] Bergwerk M Gonen T Lustig Y Amit S Lipsitch M Cohen C. Covid-19 Breakthrough Infections in vaccinated health care workers. N Engl J Med. (2021) 385:1474–84. 10.1056/NEJMoa2109072 34320281 PMC8362591

[10] Halpin SJ McIvor C Whyatt G Adams A Harvey O McLean L. Postdischarge symptoms and rehabilitation needs in survivors of COVID-19 infection: a cross-sectional evaluation. J Med Virol. (2021) 93:1013–22. 10.1002/jmv.26368 32729939

[11] LaVergne SM Stromberg S Baxter BA Webb TL Dutt TS Berry K. A longitudinal SARS-CoV-2 biorepository for COVID-19 survivors with and without post-acute sequelae. BMC Infect Dis. (2021) 21:677. 10.1186/s12879-021-06359-2 34256735 PMC8276222

[12] Moreno-Perez O Merino E Leon-Ramirez JM Andres M Ramos JM Arenas-Jimenez J. Post-acute COVID-19 syndrome. Incidence and risk factors: a mediterranean cohort study. J Infect. (2021) 82:378–83. 10.1016/j.jinf.2021.01.004 33450302 PMC7802523

[13] Lo YL. COVID-19, fatigue, and dysautonomia. J Med Virol. (2021) 93:1213. 10.1002/jmv.26552 32975809

[14] Goldstein DS. The extended autonomic system, dyshomeostasis, and COVID-19. Clin Auton Res. (2020) 30:299–315. 10.1007/s10286-020-00714-0 32700055 PMC7374073

[15] (NICE) NIfHaCE,. COVID-19 Rapid Guideline: Managing The Long-Term Effects of COVID-19. (2021). Available online at: https://www.nice.org.uk/guidance/ng188 (accessed June, 2021).

[16] Dixit NM Churchill A Nsair A Hsu JJ. Post-acute COVID-19 syndrome and the cardiovascular system: what is known? Am Heart J Plus. (2021) 5:100025. 10.1016/j.ahjo.2021.100025 34192289 PMC8223036

[17] Kalter L. Fauci introduces new acronym for long COVID at white house briefing. Medscape. (2021, February 24). Available online at: https://www.medscape.com/viewarticle/946419 (accessed April 8, 2022).

[18] Soriano JB Murthy S Marshall JC Relan P Diaz JV. A clinical case definition of Post-COVID-19 condition by a Delphi consensus. Lancet Infect Dis. (2021). 10.1016/S1473-3099(21)00703-9 PMC8691845 34951953

[19] Miglis MG Goodman BP Chemali KR Stiles L. Re: 'Post-COVID-19 chronic symptoms' by Davido et al. Clin Microbiol Infect. (2021) 27:494. 10.1016/j.cmi.2020.08.028 32891765 PMC7470728

[20] Cheshire WP Jr. Autonomic history, examination, and laboratory evaluation. Continuum. (2020) 26:25–43. 10.1212/CON.0000000000000815 31996620

[21] Romero-Sanchez CM Diaz-Maroto I Fernandez-Diaz E Sanchez-Larsen A Layos-Romero A Garcia-Garcia J. Neurologic manifestations in hospitalized patients with COVID-19: the ALBACOVID registry. Neurology. (2020) 95:e1060–e70. 10.1212/WNL.0000000000009937 32482845 PMC7668545

[22] Misra S Kolappa K Prasad M Radhakrishnan D Thakur KT Solomon T. Frequency of neurologic manifestations in COVID-19: a systematic review and meta-analysis. Neurology. (2021) 97:e2269–e81. 10.1212/WNL.0000000000012930 34635561 PMC8665434

[23] Townsend L Moloney D Finucane C McCarthy K Bergin C Bannan C. Fatigue following COVID-19 infection is not associated with autonomic dysfunction. PLoS One. (2021) 16:e0247280. 10.1371/journal.pone.0247280 33630906 PMC7906457

[24] Sandroni P Opfer-Gehrking TL McPhee BR Low PA. Postural tachycardia syndrome: clinical features and follow-up study. Mayo Clin Proc. (1999) 74:1106–10. 10.4065/74.11.1106 10560597

[25] Raj SR Arnold AC Barboi A Claydon VE Limberg JK Lucci VM. Long-COVID postural tachycardia syndrome: an American autonomic society statement. Clin Auton Res. (2021) 31:365–8. 10.1007/s10286-021-00798-2 33740207 PMC7976723

[26] Yachou Y El Idrissi A Belapasov V Ait Benali S. Neuroinvasion, neurotropic, and neuroinflammatory events of SARS-CoV-2: understanding the neurological manifestations in COVID-19 patients. Neurol Sci. (2020) 41:2657–69. 10.1007/s10072-020-04575-3 32725449 PMC7385206

[27] Rangon CM Krantic S Moyse E Fougere B. The vagal autonomic pathway of COVID-19 at the crossroad of Alzheimer's disease and aging: a review of knowledge. J Alzheimers Dis Rep. (2020) 4:537–51. 10.3233/ADR-200273 33532701 PMC7835993

[28] Cocoros NM Svensson E Szepligeti SK Vestergaard SV Szentkuti P Thomsen RW. Long-term risk of parkinson disease following influenza and other infections. JAMA Neurol. (2021) 78:1461–70. 10.1001/jamaneurol.2021.3895 34694344 PMC8546623

[29] Meinhardt J Radke J Dittmayer C Franz J Thomas C Mothes R. Olfactory transmucosal SARS-CoV-2 invasion as a port of central nervous system entry in individuals with COVID-19. Nat Neurosci. (2021) 24:168–75. 10.1038/s41593-020-00758-5 33257876

[30] Synowiec A Szczepa?ski A Barreto-Duran E Lie LK Pyrc K. Severe acute respiratory syndrome coronavirus 2 (SARS-CoV-2): a Systemic Infection. Clin Microbiol Rev. (2021) 34:e00133–20. 10.1128/CMR.00133-20 33441314 PMC7849242

[31] Bellavia S Scala I Luigetti M Brunetti V Gabrielli M Verme LZD. Instrumental evaluation of COVID-19 related dysautonomia in non-critically-ill patients: An observational, cross-sectional study. J Clin Med. (2021) 10:586. 10.3390/jcm10245861 34945155 PMC8703676

[32] Novak P Mukerji SS Alabsi HS Systrom D Marciano SP Felsenstein D. Multisystem Involvement in Post-Acute Sequelae of Coronavirus Disease 19. Ann Neurol. (2022) 9:367–79. 10.1002/ana.26286 34952975 PMC9011495

[33] Tracey KJ. The inflammatory reflex. Nature. (2002) 420:853–9. 10.1038/nature01321 12490958

[34] Gupta A Madhavan MV Sehgal K Nair N Mahajan S Sehrawat TS. Extrapulmonary manifestations of COVID-19. Nat Med. (2020) 26:1017–32. 10.1038/s41591-020-0968-3 32651579 PMC11972613

[35] Vallée A. Dysautonomia and implications for anosmia in long COVID-19 disease. J Clin Med. (2021) 10:5514. 10.3390/jcm10235514 34884216 PMC8658706

[36] Buoite Stella A Furlanis G Frezza NA Valentinotti R Ajcevic M Manganotti P. Autonomic dysfunction in Post-COVID patients with and witfhout neurological symptoms: a prospective multidomain observational study. J Neurol. (2022) 269:587–96. 10.1007/s00415-021-10735-y 34386903 PMC8359764

[37] Stanbro M Gray BH Kellicut DC. Carotidynia: revisiting an unfamiliar entity. Ann Vasc Surg. (2011) 25:1144–53. 10.1016/j.avsg.2011.06.006 22023945

[38] Finsterer J Scorza FA. Guillain-Barre syndrome in 220 patients with COVID-19. Egypt J Neurol Psychiatr Neurosurg. (2021) 57:55. 10.1186/s41983-021-00310-7 33967575 PMC8094972

[39] Seixas R Campoamor D Lopes J Bernardo T Nzwalo H Pascoalinho D. Occurrence of guillain-barre syndrome during the initial symptomatic phase of COVID-19 disease: coincidence or consequence? Cureus. (2021) 13:e19655. 10.7759/cureus.19655 34976451 PMC8678953

[40] Uncini A Vallat JM Jacobs BC. Guillain-barre syndrome in SARS-CoV-2 infection: an instant systematic review of the first six months of pandemic. J Neurol Neurosurg Psychiatry. (2020) 91:1105–10. 10.1136/jnnp-2020-324491 32855289

[41] Kajumba MM Kolls BJ Koltai DC Kaddumukasa M Kaddumukasa M Laskowitz DT. COVID-19-associated guillain-barre syndrome: Atypical para-infectious profile, symptom overlap, and increased risk of severe neurological complications. SN Compr Clin Med. (2020) 2:2702–14. 10.1007/s42399-020-00646-w 33251483 PMC7680081

[42] Biswas S Ghosh R Mandal A Pandit A Roy D Sengupta S. COVID-19 Induced miller fisher syndrome presenting with autonomic dysfunction: a unique case report and review of literature. Neurohospitalist. (2022) 12:111–6. 10.1177/19418744211016709 34950397 PMC8689536

[43] Filosto M Cotti Piccinelli S Gazzina S Foresti C Frigeni B Servalli MC. Guillain-Barre syndrome and COVID-19: an observational multicentre study from two Italian hotspot regions. J Neurol Neurosurg Psychiatry. (2021) 92:751–6. 10.1136/jnnp-2020-324837 33158914

[44] Arcila-Londono X Lewis RA. Guillain-Barre syndrome. Semin Neurol. (2012) 32:179–86. 10.1055/s-0032-1329196 23117942

[45] Karahan M Demirtas AA Hazar L Erdem S Ava S Dursun ME. Autonomic dysfunction detection by an automatic pupillometer as a non-invasive test in patients recovered from COVID-19. Graefes Arch Clin Exp Ophthalmol. (2021) 259:2821–6. 10.1007/s00417-021-05209-w 33907887 PMC8078384

[46] Vrettou CS Korompoki E Sarri K Papachatzakis I Theodorakopoulou M Chrysanthopoulou E. Pupillometry in critically ill patients with COVID-19: a prospective study. Clin Auton Res. (2020) 30:563–5. 10.1007/s10286-020-00737-7 33029750 PMC7539751

[47] Briguglio M Porta M Zuffada F Bona AR Crespi T Pino F. SARS-CoV-2 Aiming for the heart: a multicenter italian perspective about cardiovascular issues in COVID-19. Front Physiol. (2020) 11:571367. 10.3389/fphys.2020.571367 33240098 PMC7677571

[48] Kunutsor SK Whitehouse MR Blom AW Board T Kay P Wroblewski BM. One- and two-stage surgical revision of peri-prosthetic joint infection of the hip: a pooled individual participant data analysis of 44 cohort studies. Eur J Epidemiol. (2018) 33:933–46. 10.1007/s10654-018-0377-9 29623671 PMC6153557

[49] Xie Y Xu E Bowe B Al-Aly Z. Long-term cardiovascular outcomes of COVID-19. Nat Med. (2022) 28:583–90. 10.1038/s41591-022-01689-3 35132265 PMC8938267

[50] Porzionato A Emmi A Barbon S Boscolo-Berto R Stecco C Stocco E. Sympathetic activation: a potential link between comorbidities and COVID-19. FEBS J. (2020) 287:3681–8. 10.1111/febs.15481 32779891 PMC7405290

[51] Biaggioni I Shibao CA Diedrich A Muldowney JAS 3rd Laffer CL Jordan J. Blood pressure management in afferent baroreflex failure: JACC review topic of the week. J Am Coll Cardiol. (2019) 74:2939–47. 10.1016/j.jacc.2019.10.027 31806138 PMC6939863

[52] Kaufmann H Norcliffe-Kaufmann L Palma JA. Baroreflex dysfunction. N Engl J Med. (2020) 382:163–78. 10.1056/NEJMra1509723 31914243

[53] Montalvan V Lee J Bueso T de Toledo J Rivas K. Neurological manifestations of COVID-19 and other coronavirus infections: a systematic review. Clin Neurol Neurosurg. (2020) 194:105921. 10.1016/j.clineuro.2020.105921 32422545 PMC7227498

[54] Siripanthong B Nazarian S Muser D Deo R Santangeli P Khanji MY. Recognizing COVID-19-related myocarditis: The possible pathophysiology and proposed guideline for diagnosis and management. Heart Rhythm. (2020) 17:1463–71. 10.1016/j.hrthm.2020.05.001 32387246 PMC7199677

[55] Becker RC. Autonomic dysfunction in SARS-COV-2 infection acute and long-term implications COVID-19 editor's page series. J Thromb Thrombolysis. (2021) 52:692–707. 10.1007/s11239-021-02549-6 34403043 PMC8367772

[56] Al-Kuraishy HM Al-Gareeb AI Qusti S Alshammari EM Gyebi GA Batiha GE. Covid-19-induced dysautonomia: a menace of sympathetic storm. ASN Neuro. (2021) 13:17590914211057635. 10.1177/17590914211057635 34755562 PMC8586167

[57] Gonzalez-Duarte A Norcliffe-Kaufmann L. Is 'happy hypoxia' in COVID-19 a disorder of autonomic interoception? A hypothesis. Clin Auton Res. (2020) 30:331–3. 10.1007/s10286-020-00715-z 32671502 PMC7362604

[58] Mao R Qiu Y He JS Tan JY Li XH Liang J. Manifestations and prognosis of gastrointestinal and liver involvement in patients with COVID-19: a systematic review and meta-analysis. Lancet Gastroenterol Hepatol. (2020) 5:667–78. 10.1016/S2468-1253(20)30126-6 32405603 PMC7217643

[59] Manolis AS Manolis AA Manolis TA Apostolopoulos EJ Papatheou D Melita H. COVID-19 infection and cardiac arrhythmias. Trends Cardiovasc Med. (2020) 30:451–60. 10.1016/j.tcm.2020.08.002 32814095 PMC7429078

[60] Billman GE. The LF/HF ratio does not accurately measure cardiac sympatho-vagal balance. Front Physiol. (2013) 4:26. 10.3389/fphys.2013.00026 23431279 PMC3576706

[61] Davido B Seang S Tubiana R de Truchis P. Post-COVID-19 chronic symptoms: a postinfectious entity? Clin Microbiol Infect. (2020) 26:1448–9. 10.1016/j.cmi.2020.07.028 32712242 PMC7376333

[62] Lam MH Wing YK Yu MW Leung CM Ma RC Kong AP. Mental morbidities and chronic fatigue in severe acute respiratory syndrome survivors: long-term follow-up. Arch Intern Med. (2009) 169:2142–7. 10.1001/archinternmed.2009.384 20008700

[63] Lau ST Yu WC Mok NS Tsui PT Tong WL Cheng SW. Tachycardia amongst subjects recovering from severe acute respiratory syndrome (SARS). Int J Cardiol. (2005) 100:167–9. 10.1016/j.ijcard.2004.06.022 15820302 PMC7132412

[64] Ladlow P O'Sullivan O Houston A Barker-Davies R May S Mills D. Dysautonomia following COVID-19 is not associated with subjective limitations or symptoms but is associated with objective functional limitations. Heart Rhythm. (2021). 10.1016/j.hrthm.2021.12.005 PMC8656177 34896622

[65] Li H Kem DC Reim S Khan M Vanderlinde-Wood M Zillner C. Agonistic autoantibodies as vasodilators in orthostatic hypotension: a new mechanism. Hypertension. (2012) 59:402–8. 10.1161/HYPERTENSIONAHA.111.184937 22215709 PMC3275920

[66] Fedorowski A Li H Yu X Koelsch KA Harris VM Liles C. Antiadrenergic autoimmunity in postural tachycardia syndrome. Europace. (2017) 19:1211–9. 10.1093/europace/euw154 27702852 PMC5834103

[67] Li H Yu X Liles C Khan M Vanderlinde-Wood M Galloway A. Autoimmune basis for postural tachycardia syndrome. J Am Heart Assoc. (2014) 3:e000755. 10.1161/JAHA.113.000755 24572257 PMC3959717

[68] Yu X Stavrakis S Hill MA Huang S Reim S Li H. Autoantibody activation of beta-adrenergic and muscarinic receptors contributes to an “autoimmune” orthostatic hypotension. J Am Soc Hypertens. (2012) 6:40–7. 10.1016/j.jash.2011.10.003 22130180 PMC3259269

[69] Dani M Dirksen A Taraborrelli P Torocastro M Panagopoulos D Sutton R. Autonomic dysfunction in 'long COVID': rationale, physiology and management strategies. Clin Med. (2021) 21:e63–e7. 10.7861/clinmed.2020-0896 33243837 PMC7850225

[70] Ruzieh M Batizy L Dasa O Oostra C Grubb B. The role of autoantibodies in the syndromes of orthostatic intolerance: a systematic review. Scand Cardiovasc J. (2017) 51:243–7. 10.1080/14017431.2017.1355068 28738696

[71] Cutsforth-Gregory JK. Postural tachycardia syndrome and neurally mediated syncope. Continuum. (2020) 26:93–115. 10.1212/CON.0000000000000818 31996624

[72] Francois C Shibao CA Biaggioni I Duhig AM McLeod K Ogbonnaya A. Six-month use of droxidopa for neurogenic orthostatic hypotension. Mov Disord Clin Pract. (2019) 6:235–42. 10.1002/mdc3.12726 30949555 PMC6417751

[73] Palma JA Kaufmann H. Management of orthostatic hypotension. Continuum. (2020) 26:154–77. 10.1212/CON.0000000000000816 31996627 PMC7339914

[74] Baron-Esquivias G Morillo CA. Definitive pacing therapy in patients with neuromediated syncope. Lessons from the SPAIN study. Rev Esp Cardiol. (2018) 71:320–2. 10.1016/j.rec.2017.10.037 29295807

[75] Fedorowski A. Postural orthostatic tachycardia syndrome: clinical presentation, aetiology and management. J Intern Med. (2019) 285:352–66. 10.1111/joim.12852 30372565

[76] Johansson M Stahlberg M Runold M Nygren-Bonnier M Nilsson J Olshansky B. Long-Haul Post-COVID-19 symptoms presenting as a variant of postural orthostatic tachycardia syndrome: the Swedish experience. JACC Case Rep. (2021) 3:573–80. 10.1016/j.jaccas.2021.01.009 33723532 PMC7946344

[77] Thieben MJ Sandroni P Sletten DM Benrud-Larson LM Fealey RD Vernino S. Postural orthostatic tachycardia syndrome: the Mayo clinic experience. Mayo Clin Proc. (2007) 82:308–13. 10.1016/S0025-6196(11)61027-6 17352367

[78] Bryarly M Phillips LT Fu Q Vernino S Levine BD. Postural orthostatic tachycardia syndrome: JACC focus seminar. J Am Coll Cardiol. (2019) 73:1207–28. 10.1016/j.jacc.2018.11.059 30871704

[79] Olshansky B Cannom D Fedorowski A Stewart J Gibbons C Sutton R. Postural Orthostatic Tachycardia Syndrome (POTS): a critical assessment. Prog Cardiovasc Dis. (2020) 63:263–70. 10.1016/j.pcad.2020.03.010 32222376 PMC9012474

[80] Tu Y Abell TL Raj SR Mar PL. Mechanisms and management of gastrointestinal symptoms in postural orthostatic tachycardia syndrome. Neurogastroenterol Motil. (2020) 32:e14031. 10.1111/nmo.14031 33140561

[81] Goldstein DS. The possible association between COVID-19 and postural tachycardia syndrome. Heart Rhythm. (2021) 18:508–9. 10.1016/j.hrthm.2020.12.007 33316414 PMC7729277

[82] Agarwal AK Garg R Ritch A Sarkar P. Postural orthostatic tachycardia syndrome. Postgrad Med J. (2007) 83:478–80. 10.1136/pgmj.2006.055046 17621618 PMC2600095

[83] Nath A. Neurologic manifestations of severe acute respiratory syndrome coronavirus 2 infection. Continuum. (2021) 27:1051–65. 10.1212/CON.0000000000000992 34623104 PMC9527260

[84] Kanjwal K Jamal S Kichloo A Grubb BP. New-onset postural orthostatic tachycardia syndrome following coronavirus disease 2019 infection. J Innov Card Rhythm Manag. (2020) 11:4302–4. 10.19102/icrm.2020.111102 33262898 PMC7685310

[85] Bisaccia G Ricci F Recce V Serio A Iannetti G Chahal AA. Post-acute sequelae of COVID-19 and cardiovascular autonomic dysfunction: what do we know? J Cardiovasc Dev Dis. (2021) 8:156. 10.3390/jcdd8110156 34821709 PMC8621226

[86] Shouman K Vanichkachorn G Cheshire WP Suarez MD Shelly S Lamotte GJ. Autonomic dysfunction following COVID-19 infection: an early experience. Clin Auton Res. (2021) 31:385–94. 10.1007/s10286-021-00803-8 33860871 PMC8050227

[87] Hinduja A Moutairou A Calvet JH. Sudomotor dysfunction in patients recovered from COVID-19. Neurophysiol Clin. (2021) 51:193–6. 10.1016/j.neucli.2021.01.003 33551341 PMC7835104

[88] Chilazi M Duffy EY Thakkar A Michos ED. COVID and Cardiovascular disease: what we know in 2021. Curr Atheroscler Rep. (2021) 23:37. 10.1007/s11883-021-00935-2 33983522 PMC8117457

[89] Eshak N Abdelnabi M Ball S Elgwairi E Creed K Test V. Dysautonomia: an overlooked neurological manifestation in a critically ill COVID-19 patient. Am J Med Sci. (2020) 360:427–9. 10.1016/j.amjms.2020.07.022 32739039 PMC7366085

[90] Fu Q Levine BD. Exercise and non-pharmacological treatment of POTS. Auton Neurosci. (2018) 215:20–7. 10.1016/j.autneu.2018.07.001 30001836 PMC6289756

[91] Miller AJ Raj SR. Pharmacotherapy for postural tachycardia syndrome. Auton Neurosci. (2018) 215:28–36. 10.1016/j.autneu.2018.04.008 29753556

[92] Baptista AF Baltar A Okano AH Moreira A Campos ACP Fernandes AM. Applications of non-invasive neuromodulation for the management of disorders related to COVID-19. Front Neurol. (2020) 11:573718. 10.3389/fneur.2020.573718 33324324 PMC7724108

