# Supplementary material for: Dysautonomia in COVID-19 Patients: A Narrative Review on Clinical Course, Diagnostic and Therapeutic Strategies
Source: Front Neurol. 2022 May 27;13:886609. doi: 10.3389/fneur.2022.886609 (PMC9198643; doi:10.3389/fneur.2022.886609)
Supplement: Supplementary file 1 [file Table_1.DOCX]

Supplementary Material

# Supplementary Figures and Tables

**APPENDIX 1. DIAGNOSTIC CRITERIA OF POTS**

| **Diagnostic Criteria of POTS** |
| --- |
| Sustained heart rate increase of not less than 30 beats/min or above 120 beats/min within 10 min of active standing or head-up tilt.  For individuals who are younger than 19 years, the required increase is at least 40 beats/min. |
| Absence of orthostatic hypotension (i.e., sustained systolic blood pressure drop of not less than 20 mmHg). |
| Reproduction of spontaneous symptoms such as light-headedness, palpitations, tremulousness, generalized weakness, blurred vision, and fatigue. In some patients, tachycardia may trigger vasovagal syncope corresponding to spontaneous attacks in the patient’s history. |
| History of chronic orthostatic intolerance and other typical POTS-associated symptoms (for at least 6 months^a^). |
| Absence of other conditions provoking sinus tachycardia such as anxiety disorders, hyperventilation, anemia, fever, pain, infection, dehydration, hyperthyroidism, pheochromocytoma, use of cardioactive drugs (sympathomimetics, anticholinergics). |

^a^This criterion may be controversial and is not unanimously accepted as patients may seek medical advice earlier due to increasing awareness of the syndrome. However, symptoms lasting less than 3 months should be re-evaluated to confirm the diagnosis.

*This table has been endorsed by the American Academy of Neurology, the American Autonomic Society, the American College of Cardiology, the American Heart Association, the European Federation of Autonomic Societies, the European Heart Rhythm Association, the European Society of Cardiology, and the Heart Rhythm Society. Adapted from Fedorowski A. et al.* (1, 2)

**APPENDIX 2. SYMPTOMS OF ORTHOSTATIC INTOLERANCE** (3)

| **Symptoms of orthostatic intolerance** |
| --- |
| Symptoms of cerebral hypoperfusion: light-headedness, dizziness, presyncope, blurred vision, cognitive difficulties, and generalized weakness |
| Symptoms of excessive sympathoexcitation that distinguish POTS from orthostatic hypotension include palpitations, chest pain, dyspnea, tremulousness, sweating, pallor, nausea, diarrhea, and coldness of the extremities. |

**APPENDIX 3. CHARACTERISTICAL CLINICAL PRESENTATION OF POTS**

| **Cardiovascular symptoms (pathognomonic)** |
| --- |
| Main: orthostatic intolerance, orthostatic tachycardia, palpitations, dizziness, lightheadedness, (pre-) syncope, exercise intolerance  Other frequent symptoms: dyspnea, chest pain/discomfort, acrocyanosis, Raynaud phenomenon, venous pooling, limb edema |
| **Noncardiovascular symptoms (accompanying)** |
| General symptoms: General deconditioning, chronic fatigue, exhaustion, heat intolerance, fever, debility, bedridden |
| Nervous system: Headache/migraine, mental clouding (“brain fog”), cognitive impairment, concentration problems, anxiety, tremulousness, light and sound sensitivity, blurred/tunnel vision, neuropathic pain (regional), sleeping disorders, involuntary movements |
| Musculoskeletal system: Muscle fatigue, weakness, muscle pain |
| Gastrointestinal system: Nausea, dysmotility, gastroparesis, constipation, diarrhea, abdominal pain, weight loss |
| Respiratory system: Hyperventilation, bronchial asthma, shortness of breath |
| Urogenital system: Bladder dysfunction, nocturia, polyuria |
| Skin: Petechiae, rashes, erythema, telangiectasia, abnormal sudomotor regulation, diaphoresis, pallor, flushing |

*POTS = postural orthostatic tachycardia syndrome. Adapted from Fedorowski A. et al* (1), *Johansson M et al* (2) *y Parohan M et al* (4).

**REFERENCES**

1. Fedorowski A. Postural orthostatic tachycardia syndrome: clinical presentation, aetiology and management. J Intern Med. 2019;285(4):352-66.

2. Johansson M, Stahlberg M, Runold M, Nygren-Bonnier M, Nilsson J, Olshansky B, et al. Long-Haul Post-COVID-19 Symptoms Presenting as a Variant of Postural Orthostatic Tachycardia Syndrome: The Swedish Experience. JACC Case Rep. 2021;3(4):573-80.

3. Cutsforth-Gregory JK. Postural Tachycardia Syndrome and Neurally Mediated Syncope. Continuum (Minneap Minn). 2020;26(1):93-115.

4. Parohan M, Yaghoubi S, Seraji A, Javanbakht MH, Sarraf P, Djalali M. Risk factors for mortality in patients with Coronavirus disease 2019 (COVID-19) infection: a systematic review and meta-analysis of observational studies. Aging Male. 2020;23(5):1416-24.
